# Risk Assessment of the Worldwide Expansion and Outbreak of *Massicus raddei* (Blessig) (Coleoptera: Cerambycidae) Based on Host Plant and Climatic Factors

**DOI:** 10.3390/insects13080730

**Published:** 2022-08-15

**Authors:** Yufan Zhang, Yingqiao Dang, Xiaoyi Wang

**Affiliations:** 1 Key Laboratory of Forest Protection of National Forestry and Grassland Administration, Ecology and Nature Conservation Institute, Chinese Academy of Forestry, Beijing 100091, China; 2 Yunfu Forestry Bureau, Yunfu 527300, China

**Keywords:** oak longhorned beetle, pest risk analysis, climate, host plants, maximum entropy model, phytosanitary measures

## Abstract

**Simple Summary:**

The oak longhorned beetle (OLB), *Massicus raddei* (Blessig, 1872) is a well-known wood borer of oak species throughout eastern Asia, recently pest outbreaks have occurred in northeastern regions of China. Therefore, the knowledge about the global potential distribution of this borer is necessary to develop prompt monitoring or control strategies in its distribution-free areas. Here, we highlighted the global habitat suitability of OLB with host distribution and climatic factors using the Maxent model to present the worldwide potential distribution of this borer. To our best knowledge, this is the first modelling study of this trunk borer. Our results shed light on major ecological factors that shape its spatial suitability pattern, selected high risky areas threatened by this borer, provided fundamental information to take precise quarantine measures to prevent further invasion and outbreaks of this borer at the global scale.

**Abstract:**

*Massicus raddei* (Blessig) is a serious trunk borer of oak species, currently widespread only in eastern Asia. A better understanding of the invasive potential of this borer across other distribution-free areas is further needed to prevent its invasion and outbreaks. In this study, we mapped the current distribution of *M. raddei*, two susceptible hosts (*Quercus mongolia* and *Q. liaotungensis*) and all 11 host species of this borer, and then modeled their potential distributions. We comprehensively compared the current distributions and potential invasion ranges among *M. raddei*, susceptible hosts and all hosts to select areas at risk for the establishment of this borer. MaxEnt model predictions revealed that (1) the central and eastern US, a small area of central and western Europe, western Georgia, and central Argentina had suitable climates for *M. raddei*. Such highly suitable areas for this borer overlapped considerably with the current plantation and potential distributions of its hosts. Consequently, susceptible hosts and climate suitability together create the highest risk for *M. raddei* establishment and outbreaks, throughout central and eastern America, a small area of central Europe, western Norway and western Georgia, and (2) the broad host suitability across six continents creates a situation favorable for the colonization of this borer, further extending the spatial scale of possible infestation by *M. raddei* worldwide.

## 1. Introduction

*Massicus raddei* (Blessig) (Coleoptera: Cerambycidae) is a primary wood borer that mostly attacks oak species of *Quercus* and *Castanea* that are healthy or almost dying; its larvae bore into the xylem of host trees, creating large galleries that obstruct the transportation of water and nutrition, thus causing crown dieback or even death [1]. *Massicus raddei* has a wide distribution in the oriental region, including most provinces of China [2], the Korean Peninsula [3,4,5], Japan [6,7], Vietnam [8], and the Russian Far East [9,10]. Although this borer has not yet been reported on other continents or in the remaining countries of Asia [11], with reference to severe outbreaks of this borer in oak forests of northeastern China and the globalization of trade, the identification of climatically suitable areas for the invasion and permanent establishment of the borer is urgently needed. 

Due to the concealed lifestyle of wood-boring insect pests, early infestation symptoms are not clear and thus difficult to detect. Once wood borers invade distribution-free countries or regions through the transportation of seedlings, timbers or wood packing materials infested with their larvae or pupae, these nonindigenous species are more prone to bring devastating losses and are difficult to eradicate [12,13]. In the late 20th century, *Anoplophora glabripennis* and *A.* *chinensis* invaded Europe and America through pathways associated with the commercial trade of wooden packaging materials, resulting in severe economic and ecological losses [14,15]. Therefore, in the distribution-free regions of some severe wood borers, early detection and prevention measures would be more effective and resilient than subsequent management after exotic pests are introduced to avoid unprecedented damage from ecological invasion events [16,17,18]. 

In the extensive distributional areas of *M. raddei*, only forests of *Quercus mongolica* and *Quercus liaotungensis* are severely infested in Liaoning and Jilin provinces of China. Adults of these populations show highly synchronized emergence behavior every three years, while in other distribution areas, adults of this borer display annual emergence with low density and do not pose a serious threat to forestry ecosystems [2,11]. Hence, it is speculated that the appearance of a local outbreak of *M. raddei* might be triggered by particular periodical emergence behavior and the extensive distribution of the susceptible hosts *Q. mongolica* and *Q. liaotungensis* [19]. Within the same insect species, the density of periodical broods is much higher than that of nonperiodical broods, with adults appearing each year. The phenomenon is believed to be achieved through predation avoidance or predation satiation under the context of emergence periodicity [20,21]. In addition, Sun [2] reported that there were significant differences in the infestation levels of multiple hosts of *M. raddei* in the oak forests of Liaoning Province. In these damaged woodlands, the infestation rates of *Q. mongolica* and *Q. liaotungensis* were the highest, at 68.9% and 48.6%, respectively. The infestation rates of *Quercus dentata* and *Quercus aliena* were 14.1% and 13.0%, respectively, whereas other hosts of *Quercus* or *Castanea* were rarely infested. EPPO [11] also documented that the forests of *Castanea mollissima* adjacent to seriously attacked forests of *Q. mongolica* were not infested. Therefore, *Q. mongolica* and *Q. liaotungensis* should be regarded as preferred hosts, and these other hosts should be considered as moderately or poorly suitable hosts of this borer.

Common species distribution models include CLIMEX, DOMAIN, GARP, HABITAT and MaxEnt [22]. Among these modeling techniques, MaxEnt (maximum entropy model) is a relatively advantageous method for making predictions or inferences from incomplete information, such as species’ distribution data and climatic variables [23]. The MaxEnt model is characterized by high simulation accuracy, relatively simple and stable operation, and a short required temporal period [24,25]. However, the MaxEnt model seeks only to identify a suitable climate for a species and does not consider biotic interactions or dispersal ability [26]. With reference to concealed wood borers, whether a region could be suitable for their survival and establishment would be commonly determined by the hosts and climate, as hosts can provide a microclimate for most immature stages of wood borers, thus supporting possible colonization even in areas with unsuitable climatic backgrounds [18,27]. Thus, in the present study, both host and climatic factors were used to identify the potential distribution of *M. raddei* worldwide and comprehensively evaluate the risk levels of these potential areas.

Despite the harm with the high degree by this borer has been on the oak forest ecosystems of northeastern China as well as the fact that this species was recently added to the “alert list” of the European and Mediterranean Plant Protection Organization and the United States Department of Agriculture [28,29], relatively few studies have assessed the geographic dimensions of its potential as an invader. The purpose of the present study was to (i) mapped current distributions of *M.* *raddei* and its hosts and (ii) model their potential distributions based on updated presence data for this borer, susceptible hosts and all hosts. Through the integrative comparison of the suitable spatial ranges of this borer and its hosts, we further evaluated the risk levels of regions threatened by *M. raddei* globally and screened for areas that are likely to experience negative effects of this borer. These results would provide an essential component that could be used to devise proactive quarantine measures in these risky ranges.

## 2. Materials and Methods

### 2.1. Actual Distributions of M. raddei and Its Hosts

**Figure 1:**
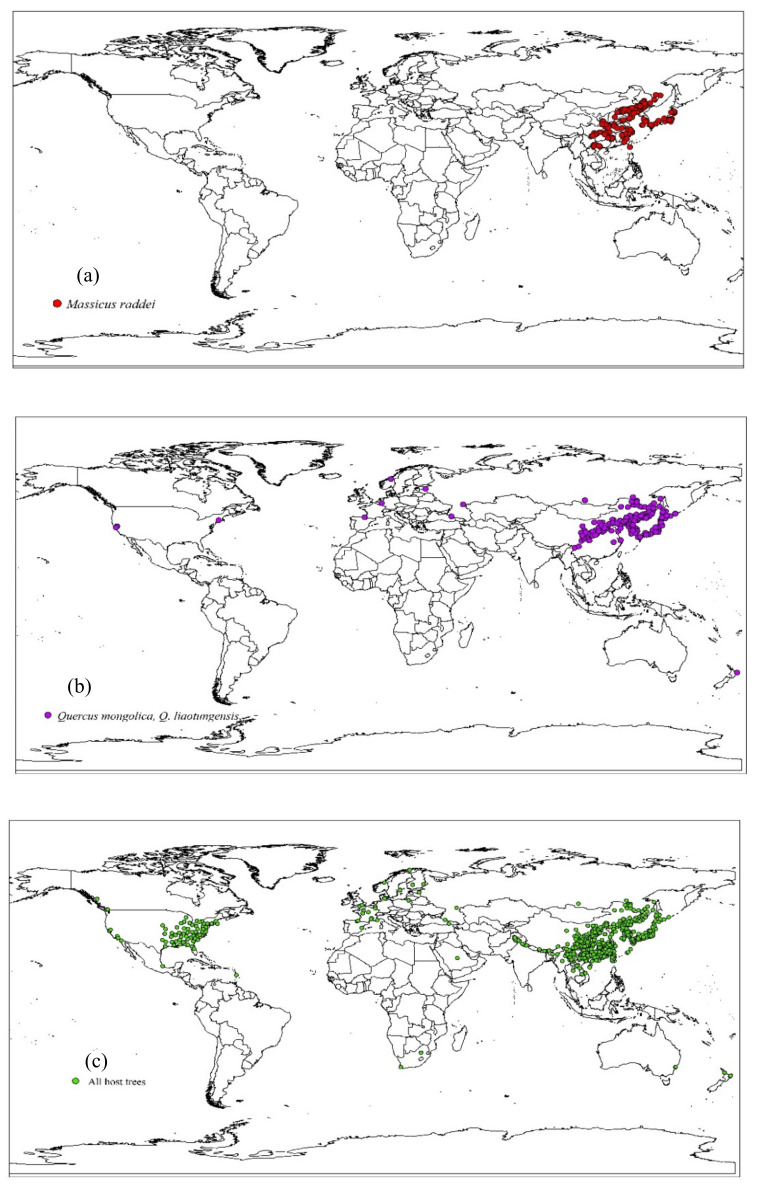
Global actual distributions of *M. raddei* (**a**), two susceptible hosts (*Q. mongolica* and *Q. liaotungensis*) (**b**) and all host trees (**c**).

In the present study, global distribution data of *M. raddei* were obtained from three sources: (1) an exhaustive literature search [2,8,9,30,31,32,33,34,35,36] (2) the Global Biodiversity Information Facility (GBIF, www.gbif.org (accessed on 20 January 2021)); and (3) field investigation and light traps in infested forests (Table S1). More than 10,000 points were initially obtained, which were sorted into Excel files based on longitude and latitude. These georeferenced coordinate points were imported into Earth Online (https://www.earthol.com (accessed on 10 February 2021)) for verification; afterward, incomplete and duplicate occurrences as well as inaccurate geographical points located at sea were manually deleted. Spatial autocorrelation is the degree of spatial association present in the datasets, and it may prevent the separation of points used for testing. Thus, spatial thinning (spThin) of species occurrence records was conducted with the spatial analyst extension of ArcGIS 10.4.1 to minimize spatial autocorrelation [37]. All remaining occurrence points were at least 10 km apart after this filtering treatment [38]. This distance ensured that each cell had only a single point of occurrence. Finally, 153 valid records of *M. raddei* were obtained and transformed into MaxEnt-compatible formats, which were used for the subsequent development of the MaxEnt model (Figure 1a).

*Massicus raddei* has been reported to commonly infest 11 host species, including *Q. mongolica*, *Q. liaotungensis*, *Q. acutissima*, *C. mollissima*, *Q. serrata*, *Q. dentata*, *Q. aliena*, *C. crenata*, *C. henryi*, *Q. glauca* and *Q. variabilis* [11]. Approximately 50,000 host distribution points were obtained through the GBIF website (accessed on 20 January 2021). Similarly, through data verification of the Earth Online and subsequent spatial filtering, 644 global host distribution points were acquired and transformed into MaxEnt-compatible formats, which were used for subsequent development of the MaxEnt model (in finally obtained presence data, two susceptible hosts, *Q. mongolica* and *Q. liaotungensis*, occupied 219 global sites) (Figure 1b,c).

### 2.2. Environmental Variables

Initially, the 19 gridded temperature and precipitation variables (1970–2000) were downloaded from the WorldClim dataset (www.worldclim.org (accessed on 20 February 2021)) with a spatial resolution of 2.5 arc minutes (ca. 5 km). This dataset has high-quality resolution, which is sufficient to determine climatic variables on a global scale [25,39].

To reduce the confounding effects of potential collinearity among environmental variables, SPSS 20.0 and ArcGIS software were used to calculate a Pearson correlation coefficient matrix between each pair of climatic variables. Pearson’s correlation coefficients higher than 0.9 were regarded as relatively strong correlations [13]. Additionally, in MaxEnt software (version 3.3.3k; www.cs.princeton.edu/wschapire/Maxent (accessed on 15 March 2021)), three preliminary models running only once were performed to predict the potentially suitable areas of *M. raddei*, two susceptible hosts and all 11 hosts by employing all 19 climatic variables to evaluate the respective influence weight of these 19 climatic variables on the possible presence of this borer or its hosts. Subsequently, only one variable per group with high similarity was included based on the contributions of the two variables to the potential presence of *M. raddei* or its hosts (the one with a relatively larger contribution rate was retained). Finally, six relatively independent environmental variables were chosen for the subsequent development of three MaxEnt projections for the potential distributions of *M. raddei*, two susceptible hosts and all 11 hosts, respectively [40] (Table 1, Table 2 and Table 3).

### 2.3. Model Development and Accuracy Evaluation

The distribution data points for *M. raddei*, susceptible hosts and all hosts as well as the corresponding dominant climatic variables were imported into MaxEnt. In the predictions of three models based on the distributional data for *M. raddei*, susceptible hosts and all hosts of this borer, 75% of the location point data were randomly selected as the training set, 25% were used to test the predictive ability of models, and other parameters were set as default values [13]. The model was performed ten times, and the average results of tenfold cross-validation were taken as the final outcome. The jackknife method was used to test the weight of single explanatory variables [23].

The accuracy of the predicted results was quantitatively assessed by the conventional metrics of the area under the receiver operating characteristic curve (AUC) and omission rate (OR). The AUC and OR values were used to measure the discriminatory ability and the overfitting levels of each model, respectively. Therein, the AUC values varied from 0 (no predictive ability) to 1 (high predictive ability), with values exceeding 0.9 indicating acceptable performance [41]; additionally, OR values ranged from OR_MTP_ (0 as expected values) and OR_10_ (0.1 as expected values). For the OR values, low performance is indicated when the values exceeded the expected values [38,42].

### 2.4. Classification and Calculation of Suitable Areas

ArcGIS software was used to extract the final MaxEnt outputs, which were associated with the invasion risk levels of *M. raddei* and the cultivation suitability levels for its hosts. Jenks natural breaks classification in the spatial analysis toolbox of ArcGIS was used to categorize the suitability levels of the potential distributions of *M. raddei*, its susceptible hosts and all hosts into four classes (unsuitable, low suitable, moderate suitable and high suitable) [13]. According to the prediction results of the potentially suitable areas, most regions with a suitable distribution for *M. raddei* and its hosts were located in China and North America. The quantization of local suitable areas of *M. raddei*, two susceptible hosts, and all hosts at three spatial scales (China, North America, and globally) will shed light on the correlation of and difference in their potential distributional dimensions worldwide. Additionally, by applying the Project Raster tool in Projections and Transformations of ArcGIS, the geographic coordinate system (GCS_WGS_1984) was transformed into the projection coordinate system (WGS_1984_Web_Mercator_Auxiliary_Sphere) to calculate the area of the regions with each of the four suitability levels throughout three spatial ranges (China, the US and Canada, and globally). The occupancy percentages of each of the four suitability levels of the potential distributions of this borer, two susceptible hosts and all 11 hosts were then plotted in Excel 2019.

## 3. Results

### 3.1. Current Distributions of M. raddei and Its Hosts

Susceptible hosts of *M. raddei* are intensively distributed in eastern China, Japan and the Korean Peninsula and are scattered in the Russian Far East, central Europe and the US. There is synchronization between the distribution of susceptible hosts and the infestation of *M. raddei* to some extent. *Massicus raddei* would find high climatic suitability throughout the extensive coverage range of two susceptible hosts (Figure 1a,b). All 11 hosts of this borer are grown on five continents (except Antarctica and South America) (including a wide area of eastern North America and eastern Asia as well as in scattered regions of western and southern North America, central Europe, eastern Oceania, and Africa) (Figure 1c).

### 3.2. Global Potential Distribution of M. raddei

**Figure 2:**
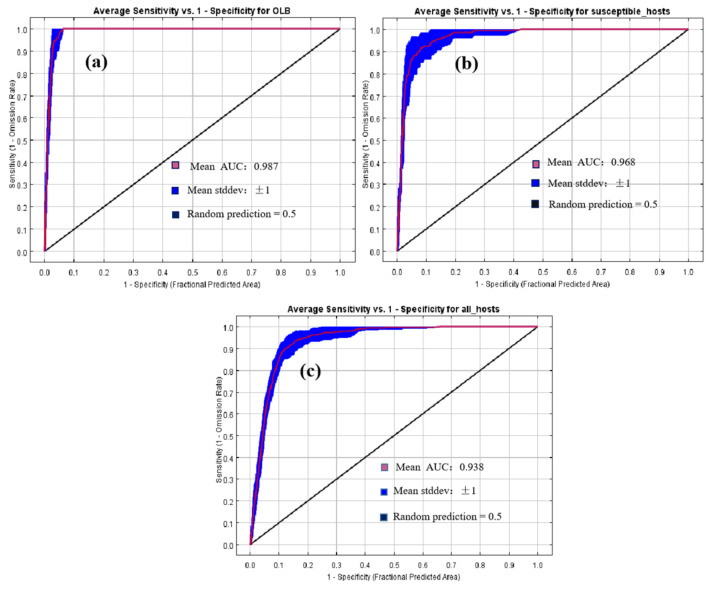
AUC values of the test on the applicability of three MaxEnt models for *M. raddei* (oak longhorned beetle, OLB) (**a**); for susceptible hosts of *M. raddei* (*Q. mongolica* and *Q. liaotungensis*) (**b**); and for all hosts of *M. raddei* (**c**).

**Figure 3:**
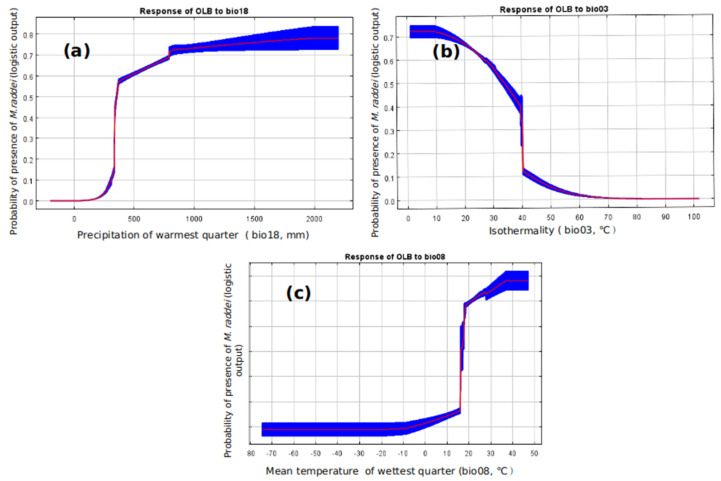
Response curves of the key climatic variables to the presence probability of *M. raddei* (OLB) in the MaxEnt model (precipitation of the warmest quarter (**a**); isothermality (**b**); mean temperature of the wettest quarter (**c**)).

The AUC_test_ value of the predicted model of the potential distribution of *M. raddei* was 0.987, indicating “excellent” prediction precision, although there was some degree of overfitting, as the OR_MTP_ value (0.02) and OR_10_ value (0.1525) were slightly higher than the corresponding expected values (Figure 2a). The precipitation of the warmest quarter (Bio_18; mm), isothermality (Bio_03, °C), mean temperature of the wettest quarter (Bio_08, °C), annual mean temperature (Bio_01, °C), temperature seasonality (Bio_04, °C) and maximum temperature of the warmest month (Bio_05, °C) were identified as the dominant environmental variables that contributed to the presence of *M. raddei*, with contribution rates of 53.6%, 25.6%, 14.5%, 4.4%, 1.1% and 0.8%, respectively (Table 1). The probability of the presence of *M. raddei* was highest at sites with a mean temperature in the wettest quarter ranging between 35 °C and 45 °C. As the Bio_08 value increased, the survival possibility of this borer progressively increased, whereas as the Bio_03 value increased, the responsive tendency of the climatically suitable likelihood of this borer decreased (Figure 3).

**Figure 4:**
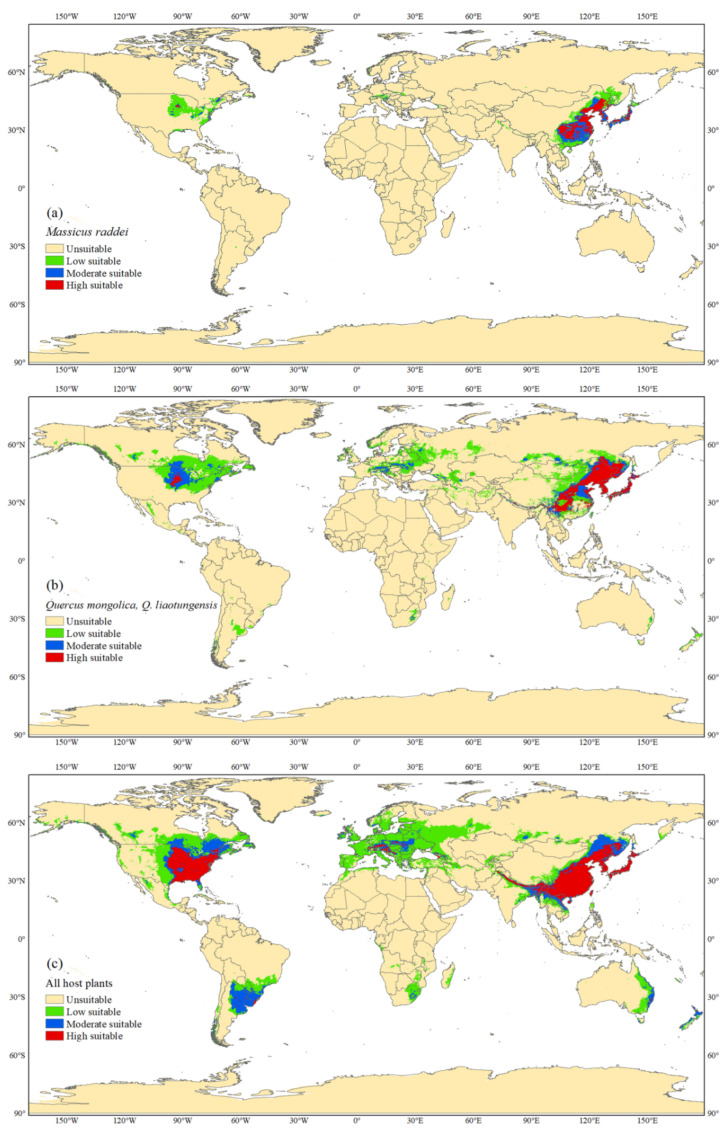
Predicted worldwide suitable habitat for *M. raddei* (**a**), susceptible hosts *Q. mongolica* and *Q. liaotungensis* (**b**) and all hosts by the MaxEnt model (**c**).

**Figure 5:**
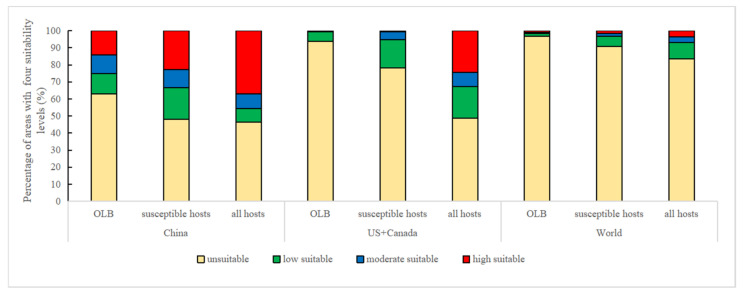
Percentage of areas of four suitability levels (unsuitability, low suitability, moderate suitability, and high suitability) for *M. raddei* (OLB), its two susceptible hosts (*Q. mongolica* and *Q. liaotungensis*) and all 11 hosts at three spatial ranges (China, US + Canada, the whole world) based on three Maxent model results.

The MaxEnt model predicted that climatically suitable areas of *M. raddei* were mainly located in the temperate continental monsoon, temperate monsoon and subtropical monsoon and monsoon humid climate zones. Globally, suitable habitats under current climatic scenarios were located in eastern China, the Korean Peninsula, Japan, the Russian Far East, the eastern US, small areas of northern Vietnam, northern India, northern Pakistan, western Georgia, southwestern Ukraine, Liechtenstein, southern Poland, southern Germany, northern Italy, western Norway, southeastern Canada, and central Argentina. Among these sites, eastern China, the Korean Peninsula, Japan and the central US showed high and medium *M. raddei* survival probabilities, and the total area of suitable regions in China and the US was approximately 4.5 × 10^6^ km^2^, accounting for approximately 99% of the overall range of suitable distribution (Figure 4a and Figure 5).

### 3.3. Global Potential Distributions of Hosts

The MaxEnt model showed a high degree of discriminatory ability to simulate the global potential suitable distributions of *Q. mongolica* and *Q. liaotungensis*, with an AUC_test_ value of 0.968. However, the OR_MTP_ value (0.0588) and OR_10_ value (0.2353) were both slightly higher than expected values, which suggested some degree of overfitting for this model (Figure 2b). The precipitation of the warmest quarter (Bio 18; mm), annual mean temperature (Bio_01; °C), maximum temperature of the warmest month (Bio_05; °C), isothermality (Bio 03; °C), precipitation seasonality (Bio 15; mm) and mean diurnal range (Bio 02; °C) were determined as the leading climatic variables affecting the presence of these two hosts, with contributions of 43.0%, 30.9%, 13.5%, 6.0%, 5.1% and 1.5%, respectively (Table 2). The spatial range of the potential distributions of the two susceptible hosts was wider than that of *M. raddei*, and there were suitable regions found on all continents except Antarctica. Throughout these suitable locations, the two susceptible hosts would have optimal climatic conditions throughout eastern Asia and the central US as well as in a small part of central Europe (Figure 4b and Figure 5).

The MaxEnt model projected the potential distribution of 11 common hosts of *M. raddei* with an AUC_test_ value of 0.938, which indicated a high level of model prediction accuracy. Both the OR_MTP_ value (0.0019) and the OR_10_ value (0.117) closely met the expected values, indicating that the model was well fitted (Figure 2). The precipitation of the warmest quarter (Bio 18; mm), isothermality (Bio 03; °C), annual mean temperature (Bio 01; °C), mean temperature of the warmest quarter (Bio 10; °C), annual precipitation (Bio 12; mm) and mean temperature of the wettest quarter (Bio 08; °C) were the strongest constraints affecting the distributions of all host species, with contributions of 49.9%, 20.7%, 13.9%, 7.9%, 6.7% and 0.9%, respectively (Table 3). The range of potential distribution for all hosts became wider than that of this borer species and its two susceptible hosts, and all continents except Antarctica had extensive regions that could be deemed suitable for the distribution and colonization of all host species. Similarly, the highly and moderately suitable regions of all hosts were concentrated in eastern Asia, eastern North America, central Europe, southern South America and a small area of eastern Oceania (Figure 4c and Figure 5).

## 4. Discussion

The present study is the first attempt to create a map illustrating the present distribution of *M. raddei* and assess the global potential invasive range of this borer. The results could help to comprehensively understand the risk levels of every country threatened by this borer at a global scale. The results also suggested that *Q. mongolica* and *Q. liaotungensis*, which have been or are being planted in Europe and the mid-eastern US, are especially at risk if this borer successfully invades and establishes in the surrounding environment. The results further emphasized the need for regular monitoring and pre-emptive quarantine measures in all focal districts to strengthen the management of imported oak timber or products, fundamentally preventing the invasion of this borer at the global scale.

The precipitation of the warmest quarter, isothermality, and mean temperature of the wettest quarter shared more than a 10% contribution rate and thus played a dominant role in the potential distribution of *M. raddei*. Given that most developmental stages of the borer are concealed in host trees, as long as the external climatic factors can satisfy the need for emergence and the reproductive activities of adults, possible adaptation and dispersal could occur in these regions. Previous studies have documented that temperature and humidity are the two essential ecological factors determining the eclosion and reproduction of *M. raddei* adults [43,44]. Tang [43] and Yang et al. [44] concluded that environmental temperatures ranging from 22 °C to 26 °C and environmental humidity values ranging from 50% to 80% were favorable for the life activities of adults of this borer species. The emergence density of adults gradually increases with increasing temperature during the emergence period, whereas the emergence performance of this species is negatively affected by high humidity [43,44]. Additionally, temperature and precipitation would have certain effects on the soil water supply, growth and timber productivity of hosts of *M. raddei*, which would indirectly impact the incidence rate of this borer [13,45,46]. Furthermore, the present study discovered that the probability of the presence of *M. raddei* tended to initially gradually increase and then remained flat with the increases in the precipitation of the warmest quarter and the mean temperature of the wettest quarter. The probability of the presence of this borer presented the trend of gradually declining and then remaining flat with the enhancement of isothermality. In general, the locations with higher altitudes were often characterized by larger temperature discrepancies between day and night, which could provide a plausible reason explaining the absence of *M. raddei* throughout some areas with high altitudes, such as the Xinjiang Uygur Autonomous Region and the Tibet Autonomous Region of China [46].

*Massicus raddei* is widely distributed in eastern Asia, but only the oak forests of Liaoning and Jilin Provinces in China are severely infested. Sun [2] and Zhang et al. [19,47] both concluded that regional outbreaks of *M. raddei* in northeastern China could be facilitated by the combined effects of particular periodical emergence behavior and the extensive distribution of susceptible host species. Hence, the present study specifically identified the current distribution and projected the potential distribution of the two susceptible hosts. Our predicted results showed that *M. raddei* and its susceptible hosts had similar ecological niches in most of the studied regions. Accordingly, the existence of *M. raddei* was always identified in regions where susceptible hosts are widely grown (Figure 1a,b). Based on the predictions of their potentially suitable areas, the range of the global potential distribution of *M. raddei* was contained in that of its susceptible hosts. Several previous studies observed similar results. For instance, Rank et al. [17] proposed that Lobesia botrana (Denis and Schiffermuller) and its host Vitis vinifera L. occupied a similar ecological niche, and all potential suitable cropping areas would experience the risk of being invaded by this pest. Gómez et al. [48] projected at-risk areas of *Labidostomis lusitanica* (Germar, 1824) across pistachio cultivation at the Iberian Peninsula level and observed that this pest would find an optimal suitable climate mainly in the areas in which pistachios also had suitable climatic conditions for their growth. In the present study, only the overlapping areas of the potential distributions of *M. raddei* and current plantation areas or the potentially suitable regions of its susceptible hosts could be reasonably considered as risky areas. In these key areas, *Massicus raddei* is most likely to develop populations and cause epidemic outbreaks once this borer is introduced into these regions and susceptible hosts are planted rapidly in common climatically favorable zones. For example, the central and eastern US, central Europe, western Norway, and western Georgia were identified as suitable areas for *M. raddei*, and susceptible hosts of this borer were distributed in the surrounding environments of these pest-free countries (Figure 1b and Figure 4a). Meanwhile, it was previously reported that *Q. mongolica* and *Q. liaotungensis* were excessively grown in Europe and the US, and the cultivation coverage was still dramatically expanding. These two susceptible hosts have also been sold and recommended for cultivation in Nebraska and North Dakota of the US [28,29,49,50,51]. These anthropogenic plantation activities would provide a great nutritional basis for the colonization and subsequent outbreak of *M. raddei*.

In this study, the prediction results of the potential distributions of 11 common hosts of *M. raddei* were determined as an essential reference for assessing the suitable spatial range of this borer. There are several reasons supporting the feasibility of this approach for this borer: (1) Compared with other groups of insects, wood borers generally have a concealed and long-term lifestyle [52]. Most developmental stages (egg, larva and pupa) of *M. raddei* are protected by the bark and wood tissues and are scarcely affected by external climatic conditions; only the transient emergence and reproduction stages of adults are exposed to the areas outside of host trees [2]. Thus, the colonization risks of undistributed areas posed by this borer species will be extended by the microclimate provided by its hosts, flexible host suitability and availability of transfer hosts. Similarly, Shim et al. [18] and Dang et al. [27] both emphasized the necessary role of hosts in delineating the potential distribution of wood borers and proposed that even if some regions showed climatic unsuitability, the occurrence and colonization of wood borers could be supported by favorable host distribution in these regions. (2) *Massicus raddei* is oligophagous, with hosts mainly concentrated on *Quercus* and *Castanea* [2,43]. Previous studies also identified several host species belonging to additional genera, whereas Luo et al. [53] proposed that the accuracy of these reported attacked hosts of this borer on species other than oak trees still needed to be verified. (3) Adults of this borer species prefer to aggregately feed on sap on the trunk cracks of oak trees to complement their nutrients before reproduction and oviposition. Larvae of this borer bore within oak trees to allow its development and survival [11]. Therefore, as long as these related hosts are widely grown, their fundamental survival prerequisites will persist. In our present study, the plantation of secondary hosts and the climatic suitability for *M. raddei* were commonly observed, throughout these distribution-free regions including the central US, southeastern Canada, northern India, northern Pakistan and northern Vietnam. This ecological situation would support potential colonization for possible invasive populations of this borer. With regard to the particular life habits of this borer, it is reasonably assumed that the borer has the potential to establish and develop populations across these large-scale regions with conditions that are suitable to the host but unsuitable to the borer itself once its hosts are widely planted. Furthermore, this borer will benefit from its strong host adaptability, facilitating its switch to attack native novel hosts of *Quercus*, *Castanea* or other genera as alternate hosts after its incursion [11,29]. The extent of host distribution of this borer is, hence, likely to be a conservative estimate to be employed in present predictions. Meanwhile, these alternate hosts in the invasion regions generally lack a coevolutionary history with *M. raddei*, which might make them more vulnerable to infestation by this borer than the original hosts. Due to lag effects of natural enemies, invaded regions generally lack suppression effects of natural enemies on different developmental stages of this borer for a long time. Under this ecological context, *Massicus raddei* will be more likely to cause destructive outbreaks once this borer invades undistributed areas [15,42,54,55].

Since the end of 2016, China has issued laws to protect all state-owned natural forests mostly centered in northeastern China. Any anthropic behaviors of tree felling need to receive official authorization by the central forestry administrations and are exclusively applicable for sanitary and scientific matters [56,57]. In light of the low migration capacity of *M. raddei*, international timber transport is one of the main pathways of its spread. Therefore, under current circumstances, borer introduction events are less likely to occur from these infested oak forests in China. However, this borer still has a large likelihood of invading and spreading through other pathways correlated with trades in private nursery stocks and wood products together with oak timber trades from countries other than China. In summary, quarantine measures should be strictly implemented, especially when timber or wood products of known hosts of *M. raddei* are imported from regions with an existing *M. raddei* population into climatically suitable areas of this borer or its hosts. Additionally, early warning and sustainable monitoring measures should be conducted across all potential distribution areas of this borer, especially for high-risk areas, including the mid-eastern US and central Europe, which could help to timely update species’ recent distributional information, properly implement or modify recent management strategies and then further eliminate or reduce the chances of invasion, colonization and epidemic outbreaks worldwide.

The MaxEnt modeling approach is most frequently applied to predict potential species distributions with great performance and has been utilized for a wide array of wood-boring insects in recent years [13,27,42,58]. However, there are some limitations weakening the prediction of the models used here. On the one hand, it is worth noting that the MaxEnt model describes a species’ fundamental niche, rather than its realized niche, of which the prediction accuracy will be based on the quantity and quality of the presence data and the appropriateness of applied environmental factors [48,59,60]. The concealed life-history characteristic of this borer makes it more difficult to detect, especially in pest populations with low density, such as most oak forests of southern China. Consequently, the presence sites of this borer provided by our study might be far less than its real condition. The actual global potential distributional range of this borer could expand or be limited when adding more presence sites and other possible climatic zones for this borer. On the other hand, climatic conditions and host availability are only one part of the ecological components that affect the possibility of species’ survival ability and shape its range. We failed to consider additional abiotic and biotic drivers of distributions in the present models (e.g., competition with other insect species, parasitism or predation by natural enemy insects, habitat type, forest structure), likely further widening or narrowing the potential range of this borer. In future studies, research on the detection and monitoring methods is needed. Additionally, it is necessary to include more distribution records, consider more ecological constraints, and apply more multiple prediction models to allow for a deeper comprehension of the potential geographical dimensions of *M. raddei* worldwide.

## 5. Conclusions

The present study used MaxEnt and ArcGIS software to project the global potentially suitable areas of *M. raddei*, its susceptible hosts and all 11 hosts based on integrative distribution data to highlight key areas susceptible to invasion by this borer species. Results showed that there was substantial potential for the inspersion of *M. raddei* into areas where its hosts have been or are being planted in central and eastern America, small areas of central Europe, western Norway and western Georgia. Meanwhile, extensive expansion potential of this borer species was identified on all continents except Antarctica (including eastern Asia, central Europe, eastern North America, small areas of southern South America, eastern Oceania and southern Africa), which was supported by the same widespread potentially suitable areas for its hosts in these regions. These results can be used as an early warning to decision-makers and allow them to implement phytosanitary measures to prevent or slow the invasion of this borer into their jurisdiction.

## Figures and Tables

**Table 1:** The accumulated contribution of selected environmental variables to the potential distribution of *M. raddei* defined by MaxEnt.

Environmental Variables	Percent Contribution/%	Accumulated Percent Contribution/%
Precipitation of the warmest quarter; Bio18	53.6	53.6
Isothermality; Bio03	25.6	79.2
Mean temperature of the wettest quarter; Bio08	14.5	93.7
Annual mean temperature; Bio01	4.4	98.1
Temperature seasonality; Bio04	1.1	99.2
Maximum temperature of the warmest month; Bio05	0.8	100.0

**Table 2:** The accumulated contribution of selected environmental variables to the potential distribution of the susceptible hosts *Q. mongolica* and *Q. liaotungensis* of *M. raddei* defined by MaxEnt.

Environmental Variables	Percent Contribution/%	Accumulated Percent Contribution/%
Precipitation of the warmest quarter; Bio18	43.0	43.0
Annual mean temperature; Bio01	30.9	73.9
Maximum temperature of the warmest month; Bio05	13.5	87.4
Isothermality; Bio03	6.0	93.4
Precipitation seasonality; Bio15	5.1	98.5
Mean diurnal range; Bio02	1.5	100.0

**Table 3:** The accumulated contribution of selected environmental variables to the potential distribution of all hosts of *M. raddei* defined by MaxEnt.

Environmental Variables	Percent Contribution/%	Accumulated Percent Contribution/%
Precipitation of the warmest quarter; Bio18	49.9	49.9
Isothermality; Bio03	20.7	70.6
Annual mean temperature; Bio01	13.9	84.5
Mean temperature of the warmest quarter; Bio10	7.9	92.4
Annual precipitation; Bio12	6.7	99.1
Mean temperature of the wettest quarter; Bio08	0.9	100.0

## Data Availability

The data presented in this study are available in the article. Distribution data points of OLB were collected from http://www.gbif.org/ (accessed on 20 January 2021), the literature, and field investigations (Supplementary Materials: Table S1 for OLB). Distribution data points of two susceptible hosts and all 11 common hosts of this borer were only collected from http://www.gbif.org/ (accessed on 20 January 2021). Climatic variables were downloaded from http://www.worldclim.org/ (accessed on 20 February 2021).

## Supplementary Materials

The following supporting information can be downloaded at: https://www.mdpi.com/article/10.3390/insects13080730/s1, Table S1: Distributional locations of *Massicus raddei* obtained from field investigations in China.
