# Supplementary material for: Risk Assessment of the Worldwide Expansion and Outbreak of Massicus raddei (Blessig) (Coleoptera: Cerambycidae) Based on Host Plant and Climatic Factors
Source: Insects. 2022 Aug 15;13(8):730. doi: 10.3390/insects13080730 (PMC9409856; doi:10.3390/insects13080730)
Supplement: Supplementary file 1 [file insects-13-00730-s001.zip › insects-1834134-supplementary.pdf]

insects MDPI

Table S1 Distributional locations of *Massicus raddei* obtained from field investigations in China

**Distributional points of *M. raddei* through field investigations**	**Longitude (°E)**	**Latitude (°N)**
Kunyu mountain, Yantai , Shandong	121.75	37.29
Fangshan silkworm field, Qixia, Shandong	120.44	37.13
Xilu temple, Hefei, Anhui	117.00	31.72
Shushan martyrs cemetery, Hefei, Anhui	117.18	31.84
Sishan forest park, Xixia, Henan	111.28	33.17
Mangdangshan nature reserve, Nanping, Fujian	118.10	26.71
Pingbian Dawei mountain, Honghe, Yunnan	103.41	22.56
Panlong forest park, Wenshan, Yunnan	104.15	23.21
